# Fatal pulmonary *Scedosporium aurantiacum* infection in a patient after near-drowning: A case report

**DOI:** 10.18502/cmm.7.4.8410

**Published:** 2021-12

**Authors:** Roya Ghasemian, Azadeh Bandegani, Firoozeh Kermani, Leila Faeli, Behrad Roohi, Elham Yousefi-Abdolmaleki, Mohammad T. Hedayati, Emmanuel Roilides, Tahereh Shokohi

**Affiliations:** 1 Antimicrobial Resistance Research Center, Communicable Diseases Institute, Mazandaran University of Medical Sciences, Sari, Iran; 2 Student Research Committee, Mazandaran University of Medical Sciences, Sari, Iran; 3 Department of Medical Mycology, School of Medicine, Mazandaran University of Medical Sciences, Sari, Iran; 4 Invasive Fungi Research Centre (IFRC), Mazandaran University of Medical Sciences, Sari, Iran; 5 Department of Internal Medicine, School of Medicine, Mazandaran University of Medical Sciences, Sari, Iran; 6 Invasive Fungi Research Center, Communicable Diseases Institute, Mazandaran University of Medical Sciences, Sari, Iran; 7 Department of Medical Mycology, School of Medicine, Mazandaran University of Medical Sciences, Sari, Iran; 8 Infectious Diseases Section, 3rd Department of Pediatrics, Faculty of Medicine, Aristotle University School of Health Sciences, Thessaloniki, Greece

**Keywords:** Antifungal susceptibility test, Amphotericin B, Invasive pulmonary infection, Near-drowning, *Scedosporium aurantiacum*, Voriconazole

## Abstract

**Background and Purpose:**

*Scedosporium* spp. is a saprophytic fungus that may cause invasive pulmonary infection due to the aspiration of contaminated water in both immunosuppressed and immunocompetent hosts.

**Case report:**

Herein, we report a fatal case of pulmonary infection caused by *Scedosporium* species associated with a car crash and near-drowning in a sewage canal. *Scedosporium aurantiacum* isolated from bronchoalveolar lavage was identified by PCR-sequencing of *β-tubulin* genes. The minimum inhibitory concentration values for amphotericin B, itraconazole, posaconazole, isavuconazole were >16 µg/ml, and >8 µg/ml for anidulafungin, micafungin, and caspofungin. Voriconazole was found to be the most active agent with a MIC of 1 µg/ml.

**Conclusion:**

This report, as the first case of pulmonary scedosporiosis after near-drowning in Iran, highlights the importance of high suspicion in near-drowning victims, prompt identification of *Scedosporium* spp., and early initiation of appropriate antifungal therapy.

## Introduction

*Scedosporium* spp. is a saprophytic fungus that may cause invasive infection under certain underlying conditions, such as cystic fibrosis, organ transplant, hematological malignancies, neutropenia, corticosteroid therapy HIV/AIDS, or aspiration of contaminated water in both immunosuppressed and immunocompetent hosts [ 1 ]. The natural environment, including wetlands, sewage, marshes, putrilage, and salt waters, is the niche of this fungus that is more common in temperate and tropical regions [ 2 ]. The taxonomy of this genus has presented significant changes, and many new species have been defined. The genus *Scedosporium* now contains 10 species: *S. aurantiacum*, *S. minutisporum*, *S. desertorum*, *S. cereisporum*, and *S. dehoogii* [ 3 ]. According to this latest classification, the *Scedosporium apiospermum* complex comprises five species: *S. apiospermum* sensu stricto, *S. boydii*, *S. ellipsoideum*, *S. fusoideum*, and *S. angustum* [ 4 ].

The small size of this organism can allow it to enter the respiratory tract easily and the bronchial tree, causing a wide range of manifestations. They may also disseminate to other organs, especially the lungs, soft tissue, paranasal sinuses, bone, and brain [ 5 ]. Lung infections after aspiration of contaminated waters in near-drowned people mainly occur by fungal microorganisms besides bacterial agents, such as *Aspergillus* spp., Mucoralean fungus species, and S. apiospermum complex. High mortality up to 50% of scedosporiosis after near-drowning usually occurs in immunocompetent host [ 6 , 7 ].

Since *S. apiospermum* complex members are highly resistant to most conventional antifungal agents, including amphotericin B and azoles, treatment of these patients is challenging [ 8 ]. Therefore, due to the lack of specific signs and symptoms and the presence of intrinsic resistance to a wide variety of antifungals, timely diagnosis and rapid identification to species level of the causing agents of scedosporiosis is very important to the management of the disease [ 9 ]. To our knowledge, this is the first case of fungal lung infection due to *S. aurantiacum* in an immunocompetent near-drowned patient in Iran.

## Case report

**Figure 1:**
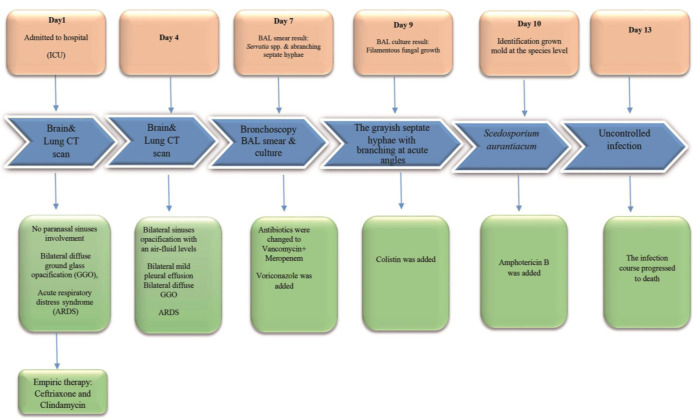
Timeline of the patient's illness

A 67-year-old woman was admitted to the ICU ward of Imam Khomeini Hospital, Sari, Iran, after a car crash and near-drowning in a sewage canal near the farmlands. The timeline of the patient's illness is illustrated in Figure 1. On the first day of admission, she was intubated and confused. Lung computed tomography (CT) showed bilateral ground-glass opacity (GGO) without pulmonary effusion compatible with acute respiratory distress syndrome (ARDS) without any involvement in the brain CT scan. There was leukocytosis with an increased neutrophil count. 

**Figure 2:**
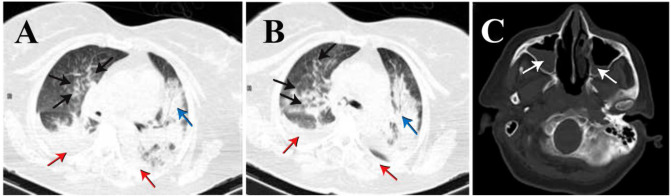
Computed tomography of the lung; Consolidation (Acinar shadow) (blue arrow) with peripheral ground-glass opacities in the lower lobe of both lung (black arrows) and bilateral mild pleural effusion (red arrows) (A and B); Computed tomography of the brain; bilateral sinuses opacification with air-fluid levels (white arrows) (C)

Sputum specimen was subjected to bacterial culture. The patient was empirically started on ceftriaxone and clindamycin. The lung CT scan on the 4th-day showed diffuse bilateral alveolar GGO in both lungs, most prominently at posterior portions. Bilateral ethmoid and maxillary sinuses opacification with air-fluid levels and calcifications of the pineal gland and bilateral choroid plexus were seen on brain CT scan (Figures 2A-C). After three days, although the cultures were negative, the antibiotics were changed to vancomycin and meropenem due to suspicion of ventilator-associated pneumonia with persistent fever. The patient was then subjected to bronchoscopy. 

**Figure 3:**
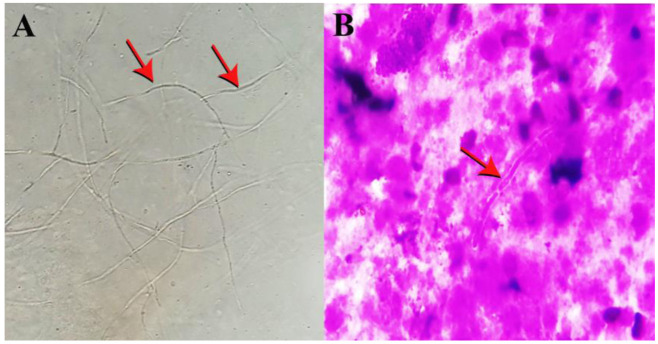
40Â microscopic view of branched septate hyaline hyphae in the KOH preparation (A) Septate hyphae view in the Giemsa stain (40Â) (B)

After homogenizing bronchoalveolar lavage (BAL) with pancreatin 0.5%, the sediment was inoculated into SABHI agar (combination of Sabouraud dextrose and brain heart infusion agar; both Condalab company, Madrid, Spain) at 30 °C for 7 days and mounted with 20% potassium hydroxide (KOH) for direct microscopic examination. In direct microscopy of BAL with a muddy-brown appearance, the grayish septate hyphae with branching at acute angles (dichotomous) were seen (Figures 3A and B). 

**Figure 4:**
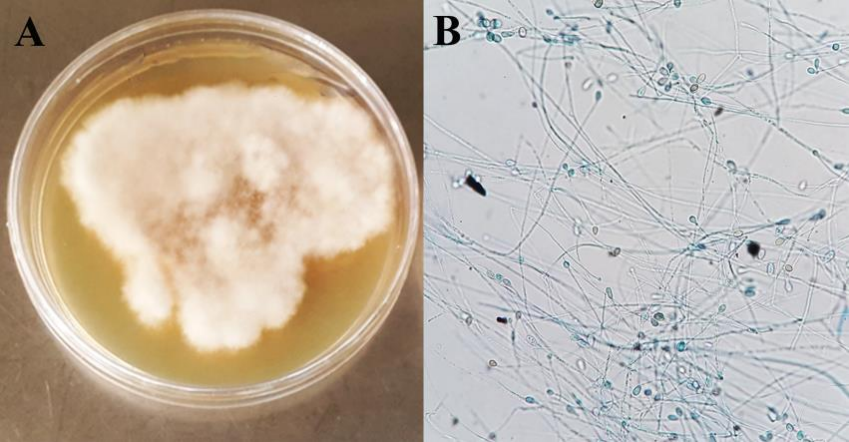
White-Gray to brown floccose colonies (A). Fungi that grew onto SDA were stained with lactophenol cotton blue: 40Â microscopic view of hyaline filamentous hyphae with oval conidia (B)

The fungal colonies were identified presumptively based on microscopic morphologic characteristics as *Scedosporium* species (Figures 4A and B). *Serratia* species were grown in BAL culture, and colistin was added to the treatment. As soon as a direct microscopy examination revealed mold infection, voriconazole IV (400 mg/day loading, then 300 mg/day) was added to the treatment regimen. The DNA of grown colonies extracted and polymerase chain reaction (PCR) assay using a set of Bt2a and Bt2b primers was performed for identification at the species level as described previously [ 3 ]. The amplicons were sequenced, compared with the GenBank database, and identified as *Scedosporium aurantiacum* with the corresponding sequences of the *S. aurantiacum* type strain CBS 101726. Afterward, the sequences were submitted to GenBank and deposited under accession number MT584762.

On the 7^th^ day of voriconazole, due to progressive infection, liposomal amphotericin B (L-AmB 5 mg/kg; 350 mg/day) was added; however, three days later, the patient died from uncontrolled infection and ARDS. In-vitro antifungal susceptibility test was performed according to M38-A2 of the Clinical and Laboratories Standards Institute. Amphotericin B, anidulafungin, caspofungin, isavuconazole, itraconazole, micafungin, posaconazole, and voriconazole were studied. Echinocandins were tested in concentrations ranging from 0.008 to 8 µg/ml, amphotericin B, and azoles from 0.016-16 µg/ml. There was high in vitro resistance to all antifungal drugs tested, except for voriconazole. The minimum inhibitory concentration values for amphotericin B, itraconazole, posaconazole, isavuconazole were > 16 µg/ml, and > 8 µg/ml for anidulafungin, micafungin, and caspofungin. Voriconazole was found to be the most active agent with MIC 1 µg/ml.

## Discussion

Due to the similarity of clinical symptoms, histopathology, and radiologic features of scedosporiosis to other filamentous fungi, such as *Aspergillus* and *Fusarium* species, diagnosis of scedosporiosis is difficult, leading to delay in the administration of appropriate antifungals and increased mortality. Therefore, it is necessary to use specific culture media, molecular techniques, and immunological tests to diagnose invasive fungal infections in near-drowned individuals [ 7 , 8 , 10 - 12 ].

The current case was an invasive lung infection due to *S. aurantiacum* that presented with bilateral pneumonia and ARDS after near-drowning. The fungus colonized the lungs after aspiration of a large amount of polluted water during near-drowning. According to these, *S. apiospermum* is the most prevalent species, and S. aurantiacum has been reported in one case in Japan [ 13 ]. *S. aurantiacum* is relatively low in Europe and China, although a high incidence was reported in Australia [ 14 ].

In the current report, *S. aurantiacum* was confirmed by sequencing the β-tubulin region with the TUB-F and TUB-R primers from the BAL culture sample. Advances in diagnostic and therapeutic modalities, such as PCR, and precise identification and susceptibility testing improvement have impacted proper and timely treatment and decreased mortality.

In our case, early ARDS was due to contaminated water aspiration and interaction between S. aurantiacum conidia with lung epithelial cells in the early stages of lung injury leading to fungal germination and epithelial cell death. Subsequently, angioinvasion and dissemination of fungi to different parts of the body may take place [ 15 ].

The same study reported that the diagnosis of *Scedosporium* infection delayed a mean of 28 days due to the lack of an available specific culture medium and the low sensitivity of routine diagnostic methods [ 7 ]. Our patient was diagnosed 10 days after admission, but she died due to uncontrolled infection and ARDS despite the prompt diagnosis and initiation of appropriate treatment. Delay in the diagnosis and timely selection of antifungal drugs could be an important reason for progressive infection leading to death. Sometimes, scedosporiosis was diagnosed post-mortem. In the studies performed by Van der Vliet et al. and Dworzack et al., similar to our study, time to diagnosis was short [ 16 ].

Treatment of scedosporiosis is still challenging, as different species of *S. apiospermum* complex have shown different susceptibility patterns against antifungal agents [ 17 ]. Results of some studies have shown that *S. aurantiacum* has the highest antifungal resistance profile among *Scedosporium* species. Results of the present study are in concordance with those of the above-mentioned studies [ 9 , 17 ]. The susceptibility of *S. aurantiacum* only to voriconazole is in agreement with previous studies [ 6 , 18 ]. Similarly, Tintelnot et al. and Gilgado et al. have reported resistance of *S. aurantiacum* to micafungin [ 9 , 18 ]. In contrast, some studies have indicated low MIC for echinocandins and azoles except for voriconazole having MIC > 8 μg/ml [ 10 ].

Despite the fact that voriconazole is considered as first-line treatment [ 19 ], several reports have shown that a combination of voriconazole, itraconazole, posaconazole, with caspofungin might be effective in the treatment after near-drowning [ 16 ]. Due to the menacing results of this infection and the effectiveness of voriconazole, using high dose voriconazole empirically is highly recommended in cases of suspected scedosporiosis or prophylactically all near-drowning victims [ 7 , 20 ].

## Conclusion

As the first case of scedosporiosis after near-drowning in Iran, this case report highlights the importance of high suspicion of *Scedosporium* infection, prompt identification of causative agents, and early initiation of appropriate antifungal therapy in near-drowning individuals.

## Acknowledgement

This study was supported by the Mazandaran University of Medical Science, which the authors gratefully acknowledge, and also we would like to thank Javad Javidnia for his critical assistance.

## Authors’ contribution

R.G., TS and M.T.H conducted the study. A.B., F.K., L.F., and B.R. made morphological, molecular analyses, and antifungal susceptibility tests of the isolated strain. R.G. and E.Y.A. collected clinical data. A.B., F.K., E.R., and T.S. created the final draft of the manuscript. All authors read and approved the final manuscript. 

## Conflict of Interest

The authors declare that there was no conflict of interest in this study.

## Financial disclosure

No financial interests related to the material of this manuscript have been declared.

## Ethical Considerations

This study was approved by the Ethics Committee of Mazandaran University of Medical Sciences, Sari, Iran (IR.MAZUMS.REC.1400.10266) and performed in compliance with the Declaration of Helsinki. Written informed consent was obtained from the legal guardians regarding the inclusion of details in the manuscript and their publication.
